# Where COVID-19 testing is challenging: a case series highlighting the role of thoracic imaging in resolving management dilemma posed by unusual presentation

**DOI:** 10.11604/pamj.2020.37.284.26697

**Published:** 2020-11-30

**Authors:** Ademola Joseph Adekanmi, Lateef Ayodele Baiyewu, Babatunde Ebenezer Osobu, Omolola Mojisola Atalabi

**Affiliations:** 1 Department of Radiology, College of Medicine, University of Ibadan, Ibadan, Nigeria,; 2 Department of Radiology, University College Hospital, Ibadan, Nigeria,; 3 Division of Cardiothoracic Surgery, Department of Surgery, College of Medicine, University of Ibadan, Ibadan, Nigeria

**Keywords:** COVID-19, surge, diagnostic challenges, viral PCR testing, thoracic imaging

## Abstract

The COVID-19 pandemic remains an evolving disease posing a challenge of incomplete understanding escalated by random atypical clinical presentations. Numerous challenges still exist with accessibility and availability of standard COVID-19 viral testing using real-time Polymerase Chain Reaction (RT-PCR), in low- and middle-income countries, especially in several hospital settings. The clinical information of three select patients at a major health facility in Southwestern Nigeria with unusual COVID-19 clinical presentation and clinical management dilemma related to challenges with COVID-19 viral laboratory testing, were retrospectively reviewed. The medical history in all three cases closely mimicked that of other medical conditions because of assumptions created by red herrings like an acute exacerbation of an underlying non-communicable disease (diaphragmatic eventration) in case 1, re-activation of a previously treated lung condition (tuberculosis) in case 2 and a sequalae of a previously diagnosed but poorly-managed chronic non-communicable disease (decompensated hypertensive heart disease). Also, viral testing was challenging in all cases due to reasons ranging from late turn-around time to inconsistent results. However, thoracic imaging was employed in all cases to heighten suspicion of COVID-19 infection, resolve management dilemma and limit intra-hospital spread. Thoracic imaging can play a major role within hospital settings in low-and middle-income countries in resolving diagnostic challenges of atypical COVID-19 clinical presentations, raising suspicion for early institution of intra-hospital disease containment measures, limiting exposure among hospital staff and guiding clinical case management of COVID-19; especially where challenges with confirmatory viral testing remain persistent.

## Introduction

Coronavirus disease 2019 (COVID-19) is an infectious disease caused by severe acute respiratory syndrome coronavirus 2 (SARS-CoV-2), a strain of coronavirus previously known as the 2019 novel coronavirus (2019-nCoV) [1,2]. Since the World Health Organization (WHO) designated this disease a pandemic of global proportions on March 11^th^ 2020, its transmission has rapidly progressed from international travel-related spread to a community spread within countries encumbered by difficulty in effecting a comprehensive public health control which has snow-balled to a recent second wave that appears to be ongoing in some western countries [3,4]. Equally, there has been a community surge in COVID-19 cases within several low- and middle-income countries (LMIC) already grappling with poor health care system set-up, ineffective public health containment measures and grave immediate and possibly long-term economic implications of the pandemic [5,6]. While the epidemiology and pathology of the disease are fairly well defined, the clinical manifestation remains dynamic and not fully understood till date.

Widely considered vital to accurate diagnosis of this disease that has a considerable number of random atypical clinical presentations, has been viral laboratory testing (VLT) employing SARS-CoV-2 real-time Polymerase Chain Reaction (RT-PCR) testing of nasal and throat swabs. Several challenges still exist in the way of deploying RT-PCR on a large scale at many LMICs, for general public infection prevention and control (IPC) [6]. Many COVID-19 RT-PCR screening centers only screen select patients with a constellation of clearly defined symptoms, given the very limited and scarce screening tools and resources in these LMIC. Therefore, it has become expedient to evolve a veritable diagnostic recourse in such testing-challenged settings, that would substantially heighten clinical suspicion of COVID-19 infection very early in the admission period of the patients, thereby resolving the dilemma associated with atypical clinical presentations and in the process, mitigate disease transmission among front-line health care workers (HCW) and by extension, the larger community that they serve.

## Methods

The clinical information of three unique cases managed at a major health facility in Southwestern Nigeria, University College Hospital, Ibadan, Nigeria; was retrospectively reviewed. The peculiar scenario used to select the cases reviewed included atypical pattern of clinical presentation of COVID-19 and the presence of management dilemma related to challenges of obtaining confirmatory real-time Polymerase Chain Reaction (RT-PCR) COVID-19 test.

Specifically, in each of the three selected case scenarios, the review focused on the atypical features of their medical history and clinical examination, presence and nature of any underlying medical condition, findings on diagnostic investigations especially serial chest X-rays, chest computed tomographic scan and naso- and oropharyngeal swab real-time Polymerase Chain Reaction (RT-PCR) COVID-19 test results; and identification of any management dilemma and rationale behind clinical decision making to resolve the situation.

The COVID-19 viral real-time Polymerase Chain Reaction (RT-PCR) testing challenges were evaluated considering the transmission dynamics and overall national response in the country at the time of each patient´s presentation to the hospital. Lengthy result turn-around time associated with low laboratory capacity was peculiar in the period when there were a few clusters of cases in the country in early April and mid-May 2020 when cases 1 and 2 presented. Case 3 was managed in early July 2020 at the community transmission phase of the infection in the country. At this stage, laboratory testing capacity had improved but intermittent shortage of test kits was still prevalent in many parts of the country.

## Results

**Patients and descriptions:** the patients were all males, aged 45 to 80 years. A case-by-case review of the three scenarios is detailed below.

**Scenario 1:** a 67 year old man, a known hypertensive and newly diagnosed diabetic inpatient, developed an intermittent left chest pain, 3 days after surgery for a locally-advanced pelvic tumour which was performed in the Trendelenburg´s position to improve intraoperative access. Pre-operatively, he had a background history of an asymptomatic left diaphragmatic eventration discovered incidentally during a routine health screening conducted 20 years previously. He had no fever, frequent sneezing, cough, abdominal pain or swelling but developed a progressive shortness of breath with poor oxygen saturation (ranging from 90-93% in room air) over the next three days after surgery. At the time, he was on routine chemoprophylaxis for venous thromboembolism. He arrived for the surgical operation from one of the COVID-19 endemic states in the country at the time, but reported no contact with any active COVID-19 case and so with the disease not yet at the community transmission stage during that period, there was no reasonable suspicion of COVID-19 infection. As well, there were only a few clusters of cases of the disease existing in the country at this time mostly related to external arrivals from western countries but his most recent foreign travel was over a year earlier.

**Figure 1:**
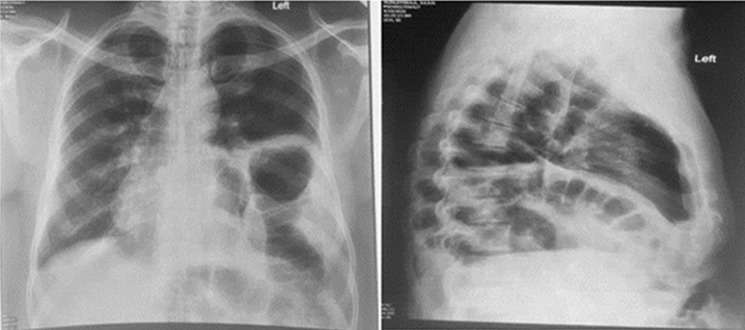
anteroposterior and lateral chest radiographs showing thinned out and markedly elevated left hemidiaphragm (diaphragmatic eventration) with mass effect of the sub-phrenic abdominal contents causing ipsilateral left lung collapse and contralateral trachea-mediastinal shift (tension viscero-thorax)

The major remarkable finding on clinical examination was tachypnoea (respiratory rate of 28/minute) with a marked reduction in breath sounds over most of the middle and lower areas of the left hemithorax with background audible bowel sounds. Initial laboratory panels revealed normal complete blood count, elevated D-dimers of 24mcg/ml (normal: <0.5mcg/ml) and mild elevation in serum creatinine of 1.8mg/dl (normal: <1.5mg/dl). Other serum electrolytes were within normal limits. Serial chest radiographs done over three days consistently revealed a thinned out and markedly elevated left hemidiaphragm (diaphragmatic eventration) with mass effect of the sub-phrenic abdominal contents causing ipsilateral left lung collapse and contralateral tracheo-mediastinal shift (tension viscero-thorax). In comparison to the pre-operative chest radiograph, the degree of eventration appeared to have increased as well as the pressure effect. Figure 1 below was the last in the series of these chest radiographs. In addition, lower limb veno-arterial duplex scan revealed early peripheral arterial disease with normal venous drainage and no evidence of deep venous thrombosis.

**Figure 2:**
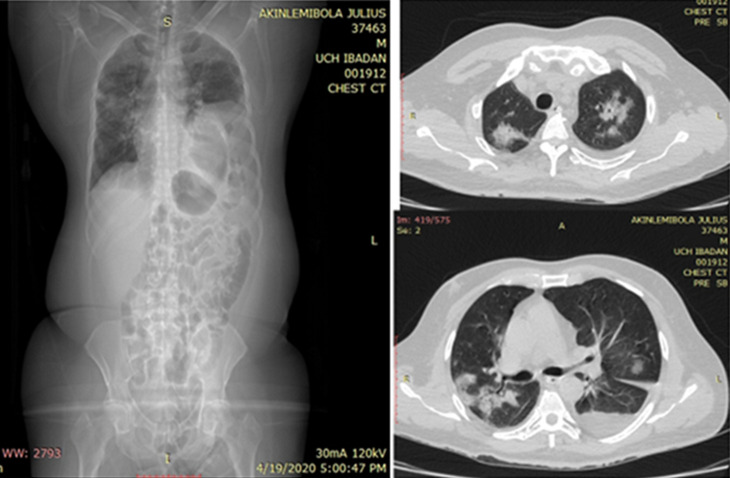
anteroposterior chest radiograph showing dextrocardia and features of bilateral pneumonic consolidations with peripheral disposition and an age-related aortic atherosclerotic calcification

**Figure 3:**
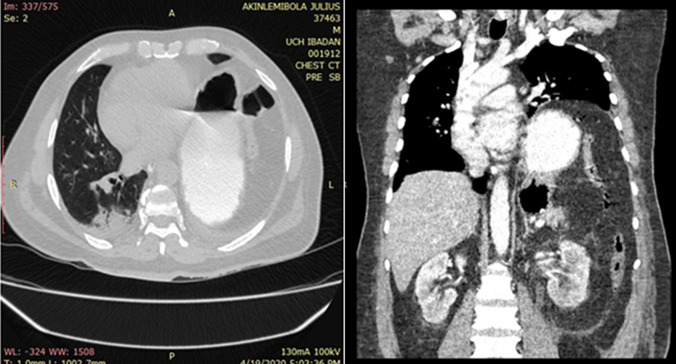
scanogram and axial views of the chest CT scan showing diffuse bilateral peribronchovascular ground glass densities and focal lung consolidation (red arrows) and a mild left pleural effusion (blue arrow)

The working diagnosis was acute exacerbation of a previously asymptomatic left diaphragmatic eventration complicated by tension viscero-thorax while pulmonary thrombo-embolism was a differential diagnosis. He was nursed head-up with supplemental oxygen support. He also had institution of full anticoagulation with enoxaparin at 1mg/kg/day dosing. However, the left chest pain progressed from an intermittent nature to a more persistent and intolerable form accompanied by deterioration in his clinical parameters {oxygen saturation dropped below 90% with persistent tachycardia (pulse rate >110/minute), tachypnoea respiratory rate >30/minute and low-grade fever (up to 38°C)}. Repeat laboratory panels revealed white cell count of 14,800 cells/μl with relative lymphopenia (5% lymphocyte differential count) and elevated C-reactive protein of 105.7 mg/L. At that point, strong consideration was given to the possibility of bowel strangulation underneath the eventrated hemidiaphragm with need for expeditious surgical management. A chest Computed Tomographic (CT) scan was performed (Figure 2, Figure 3) on the same day while awaiting the pre-operative laboratory test results, which showed the previously known left diaphragmatic eventration and its mass effect as well as multiple ground-glass densities with surrounding pneumonic consolidations diffusely aligned within both lung fields but worse in the right lower lobe. These were not visible on the chest radiograph done earlier on the same day (Figure 1). The appearance was also not consistent with early features of diffuse lymphogenous pulmonary metastasis attributable to the recently operated pelvic tumour.

**Figure 4:**
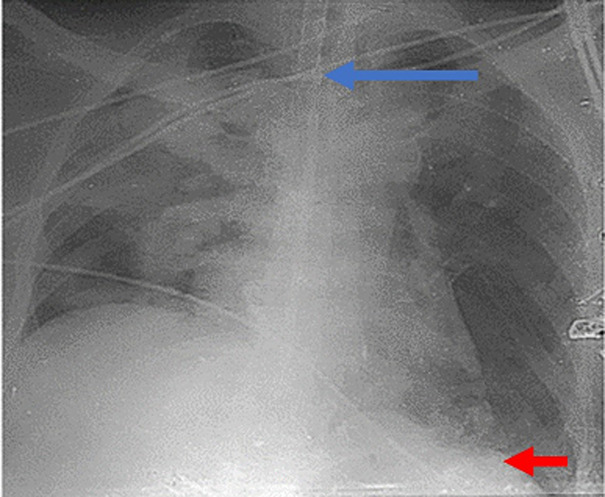
axial and coronal views of the same chest CT scan showing the eventrated left hemidiaphragm (red arrow) and tension viscero-thorax from gastric and colonic subphrenic abdominal components (blue arrow)

The chest CT scan findings heightened the suspicion of COVID-19 and nasopharyngeal and throat swabs were obtained for COVID-19 RT-PCR test. Containment measures were immediately commenced with all attending staff adequately provided with COVID-19 protective devices. He was taken to the operating room for an emergency exploratory laparotomy for the plication of the left hemidiaphragm after reduction of the stomach, transverse colon, a long segment of small bowel and the spleen; all of which were still viable. He had delayed recovery from anaesthesia necessitating elective post-operative mechanical ventilation (synchronised intermittent mandatory ventilation with pressure control mode) in the Intensive Care Unit (ICU) but he maintained poor oxygen saturation (88-93%) despite a high fraction of inspired oxygen (FiO_2_) of 1. He also had a high-grade fever (peak temperatures of 39.8°C) despite ongoing broad-spectrum systemic antibiotic, corticosteroid therapy and maintenance of therapeutic dose of enoxaparin. Post-operative chest radiograph (Figure 4) showed correct positioning of the left hemidiaphragm, return of the mediastinum and trachea to the centre and re-expansion of the left lung. The pulmonary infiltrates were more apparent at this time in the right lung field compared to the pre-operative radiographs.

The result of the COVID-19 test returned positive after 5 days by which time the patient´s clinical condition had deteriorated significantly with spiraling hyperglycemia unresponsive to insulin therapy. Surveillance and contact tracing were set in motion by institutional mechanisms and local protocols for COVID-19 infection control. Hydroxychloroquine, zinc sulphate and azithromycin combination was immediately institute based on the prevailing WHO guideline for COVID-19 case management of severe form of the disease at the time, but the patient suffered an irreversible cardiopulmonary arrest from respiratory failure within 24 hours of obtaining the test result.

**Scenario 2:** an 83 year old elderly man presented to our hospital with low grade fever and productive cough of one-week duration and shortness of breath of 3 days duration. He also reported an episode of streaky hemoptysis and a background history of treatment for a sputum-confirmed pulmonary tuberculosis, 19 years previously. There was a recent history of a self-limiting sore throat and generalized body weakness. He was hypertensive and diabetic but reported good drug compliance. He had presented at the early stage of community transmission of COVID-19 infection within the country with strong consideration given to its likelihood. However, he had been screened for COVID-19 through RT-PCR test from nasopharyngeal and throat swabs obtained at an accredited outside laboratory which returned negative; the result of which he made available at the time of admission to our facility. The remarkable findings on clinical examination were tachypnoea (respiratory rate of 36/minute), poor room air oxygen saturation of 90%, tachycardia (pulse rate of 100/minute) and systemic hypertension (blood pressure of 165/96mmHg). All initial laboratory blood panels were within normal limits except for borderline elevation in serum creatinine of 1.6mg/ml (normal: <1.5mg/dl) and low estimated glomerular filtration rate of 45.5ml/min/1.73m^2^ (normal: >90ml/min/1.73m^2^) indicating low renal reserve.

**Figure 5:**
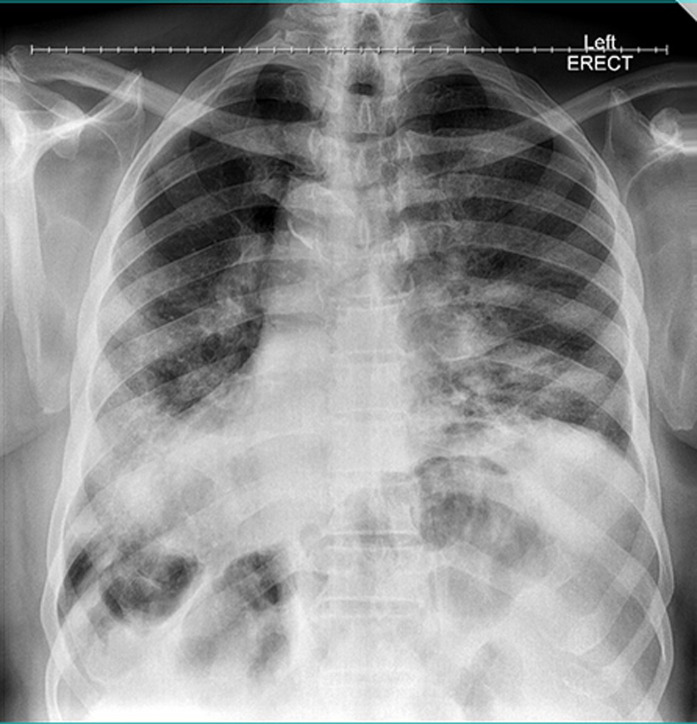
post-operative chest radiograph shows normal position of the left hemidiaphragm (red arrow) and normal position of the trachea and mediastinum (blue arrow) with right lung infiltrates

The chest radiograph done on admission revealed dextrocardia with features of multiple consolidations in both lung fields demonstrating peripheral disposition. There was no evidence of pleural or pericardial effusion (Figure 5). A 12-lead electrocardiogram revealed features of left ventricular hypertrophy and left axis deviation with sinus tachycardia. Two-dimensional trans-thoracic echocardiography revealed concentric left ventricular hypertrophy with good biventricular systolic function (left ventricular ejection fraction {EF} of 65% and Tricuspid Annular Plane Systolic Excursion {TAPSE} of 2.5cm).

**Figure 6:**
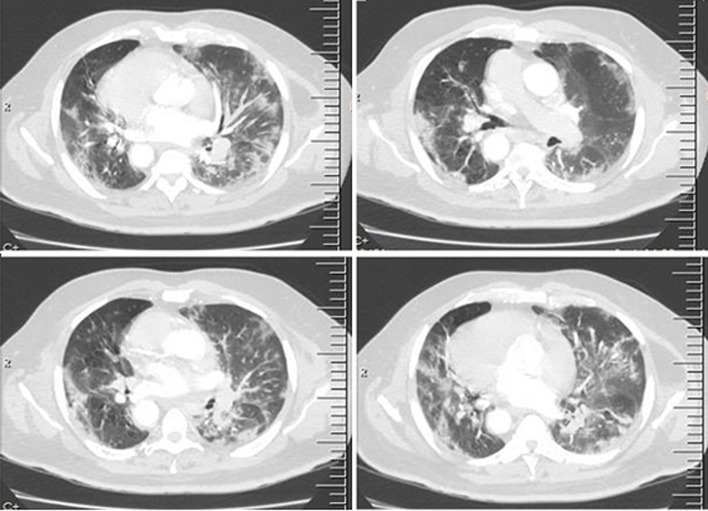
axial CT scan (lung window) showing dextrocardia, bovine pattern of aorta and diffuse peripherally and basally distributed ground-glass densities with consolidations in both lungs

Given the negative COVID-19 test, the clinical diagnosis of community-acquired bacterial pneumonia was made with a differential diagnosis of reactivated pulmonary tuberculosis. He was commenced on broad-spectrum antibiotic therapy according to hospital infectious disease protocol for community-acquired pneumonia as well as other supportive therapy, including corticosteroids and anticoagulants. Consideration was also given to commencement of a trial of empirical anti-tuberculous therapy because of his past medical history. However, his clinical condition remained poor, necessitating further imaging evaluation with chest computed tomographic (CT) scan on the 5^th^ day of admission (12^th^ day from the onset of illness) which revealed diffuse ill-defined ground glass densities in both lungs with multiple peripherally-distributed non-enhancing peri-bronchovascular consolidations especially in the lower lobes. There was bilateral pleural thickening with mild cardiomegaly (CTR=54%), rightward cardiac apex, and bovine branching pattern of a rightward aortic arch consistent with dextrocardia. The liver was located on the left and the spleen on the right, in keeping with a situs inversus (Figure 6). These imaging findings heightened suspicion of COVID-19 viral pneumonia, especially as there was a prevailing surge in community-transmitted cases at the time. The patient was transferred to the hospital isolation facility with repeat throat and nasal swabs sent for the COVID-19 test, which returned positive after five days. The patient made a full recovery from the infection and was discharged home after 16 days in the isolation centre after two subsequent consecutive COVID-19 RT-PCR tests had returned negative. No hospital staff among the primary contacts of this patient was reported to be infected with COVID-19 after contact tracing and isolation.

**Scenario 3:** a 45 year old gentleman had reported to our hospital with progressive exercise intolerance and shortness of breath of 2 weeks duration. There was an intermittent cough, productive of mucoid sputum, but he had no fever or sore throat. He also had occasional palpitations, orthopnoea and mild but progressive bilateral leg swellings. He was diagnosed with hypertensive heart disease three years previously but was poorly-compliant with prescribed medications. He presented when community transmission of COVID-19 was present in the state where the hospital is located but had reported no contact with any confirmed or suspected case. The remarkable findings on clinical examination include mild bilateral ankle oedema, tachypnoea (resting respiratory rate of 28/minute and 36/minute with minimal activity), room air oxygen saturation of 89-92% with activity-related deterioration, tachycardia of 110-118/minute pulse rate with a regular rhythm, bilateral basal lung crepitations and tender, mild hepatomegaly with no demonstrable ascites.

His initial laboratory panels revealed elevated white cell count of 13,400 cells/μl (normal: 4000-11,000 cells/μl) with relative lymphopenia of 9% differential count (normal: 25-40%). Serum D-dimer was elevated at 10mcg/ml (normal <0.5mcg/ml) as well as C-reactive protein (CRP) of 74mg/L (normal < 10mg/L). Twelve-lead electrocardiogram revealed left ventricular hypertrophy and left axis deviation with sinus tachycardia. The 2-dimensional trans-thoracic echocardiography revealed concentric left ventricular hypertrophy with borderline left ventricular ejection fraction (EF) of 55% and grade I diastolic dysfunction. The TAPSE was 1.8cm.

**Figure 7:**
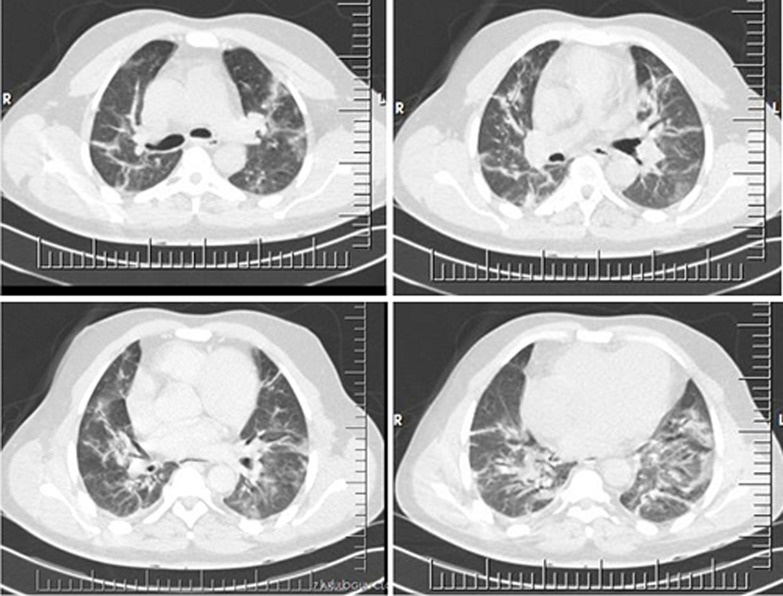
axial CT scan (lung window) showing diffuse peripherally and basally-distributed ground-glass densities with consolidations in both lungs

The diagnosis of hypertensive heart disease with decompensated biventricular cardiac failure was made with a differential diagnosis of community-acquired bacterial pneumonia. He was admitted and commenced on anti-failure medications consisting of diuretics, antihypertensives, anticoagulants and supportive therapy, especially oxygen supplementation. He had initial clinical improvement, but the shortness of breath persisted on mild to moderate exertion. He had a chest computed tomographic (CT scan) done on the 2^nd^ day of admission (Figure 7), which revealed diffuse peripherally-oriented ground glass densities in both lungs, which were more marked in the lower lobes. This heightened the suspicion of COVID-19 viral pneumonia and swab samples were sent for COVID-19 RT-PCR, which returned positive after seven days. In the interregnum, he was transferred to the hospital's isolation ward for supportive treatment and commenced on COVID-19 treatment in line with the hospital protocol. He did not require any further treatment beyond what was already instituted when the test result became available as his clinical condition had become stable.

The patient also made a full recovery after 12 days in the infectious disease isolation facility. Except for a close caregiver who also tested positive but remained asymptomatic while in isolation, no hospital staff among the primary contacts of the patient was reported to have tested positive to COVID-19.

## Discussion

The global profile of the COVID-19 viral infection has demonstrated a progressive change in the epicenter from China at the onset, to western Europe (Italy and Spain precisely) at the initial peak and later to North-America (United States of America) before the recent surges recorded in Latin America (especially Brazil). Currently, there is a growing anxiety about a second wave of the global pandemic suggesting the presence of a rapidly changing disease dynamics. Therefore, there is heavy reliance on new insights gained from patients and meticulous scientific researches that are underway in different countries of the world at this time. Indeed, with the World Health Organization (WHO), frequently providing a global direction and data-driven guideline for disease surveillance, transmission control and case management; it has become imperative that developing countries grappling with the socio-economic and clinical sequalae of the pandemic begin to provide evidence for local adaptation of global protocols to meet peculiar demands of their environments at this time [3].

From available literature, the typical and common pattern of clinical presentation of COVID-19 infection is fairly well documented and includes low grade fever, productive cough, loss of taste and smell, fatigue and shortness of breath [6]. Other less common manifestations are myalgia/arthralgia, headache, sore throat, chills, pleuritic chest pain, nausea, vomiting, nasal congestion, diarrhea, palpitations, chest tightness, conjunctivitis, self-limiting skin diseases and haemoptysis [7-10]. However, several reports from different countries indicate infrequent occurrence of atypical presentations of the infection [11-15]. The three case scenarios presented here, demonstrated unclear and atypical presentations mimicking other explainable clinical conditions like acute exacerbation of a pre-existing long-standing condition (diaphragmatic eventration) in the first scenario, a reactivation of a prevalent chronic condition (pulmonary tuberculosis) in the second scenario and an acute decompensation of a chronic poorly-managed medical condition (heart failure) due to poor drug compliance, in the third scenario. Therefore, the non-response to instituted disease-targeted medical treatment, created both diagnostic and treatment dilemma requiring early resolution. The benefit of early foreclosure of an accurate diagnosis is best appreciated when one considers that the COVID-19 infection is a highly contagious clinical condition that has a potential for rapid transmission and case explosion. It means, letting one single case slip through the keen lens of early detection and rapid case management, carries with it enormous and grave implications for infection control particularly in low-and middle-income countries (LMIC) with already stretched diagnostic and therapeutic resources [4,5].

In many countries including LMICs, COVID-19 viral laboratory testing (VLT) of throat and nasal swabs remains the most utilized platform for disease detection or confirmation [16-18]. Despite the proven efficacy of real-time Reverse Transcriptase Polymerase Chain Reaction (RT-PCR) of nasopharyngeal and throat swabs for COVID-19 diagnosis, its deployment in LMICs is still challenged by certain factors including but not limited to low testing capacity due to economic constraints and affordability; availability issues from widespread shortage of testing kits due to global market forces of demand and supply; very lengthy test result turn-out times and scarcity of cheaper and cost effective alternative testing platforms [19,20]. The cumulative effect of this challenging situation has resulted in inability to effectively coordinate a foolproof containment strategy within healthcare settings which will protect front-line healthcare workers (HCW) and disrupt the chain of transmission particularly in with these not so uncommon scenarios of atypical clinical presentations of COVID-19 [21,22].

Thoracic imaging in form of plain chest radiography/ X-ray and chest computed tomographic scanning (CT) appears to have proven diagnostic and screening relevance in COVID-19 pneumonia among patients requiring hospitalization or hospital-based care [23,24]. Though, chest radiography remains the first-line imaging modality used in the radiological evaluation of patients with suspected COVID-19 infection, it however has a far lower sensitivity when compared to chest CT scan and is further limited by the fact that abnormalities often become clearly apparent at about 10-12 days after the onset of symptoms, making it less reliable in early disease [23]. This shortcoming of chest radiography was clearly demonstrated in the first scenario where serial chest radiographs showed no remarkable consolidation and pointed instead to worsening of a previously established left diaphragmatic eventration. On the other hand, chest CT scan has proven to be reliable in the diagnosis of early and late COVID-19 pneumonia or other pulmonary complications with the typical findings including ground-glass densities (bilateral, subpleural, peripheral), crazy paving appearance (ground-glass densities and inter-/intra-lobular septal thickening), air space consolidation, bronchovascular thickening in the lesion and traction bronchiectasis [25,26]. The pattern of peripherally-oriented ground-glass lung densities with diffuse peribronchovascular consolidations was demonstrated in all three scenarios which heightened suspicion of ongoing COVID-19 viral lung infection. This contributed in no small way to changing the treatment course (as discussed in the first scenario), requesting confirmatory tests as reported in all three scenarios and commencing early containment protocol as reported in scenarios two and three.

Even in developing and LMICs, computed tomography scanners are becoming commonplace especially in large cities and urban areas [27,28], but the cost is too exorbitant for most patients. Therefore, effective deployment of serial chest X-ray at days 8 - 12 of the disease and selective chest CT examination for those that could afford it could resolve most diagnostic dilemma in uncommon situations or escalate suspicion and guide disease surveillance and transmission control, with far reaching benefits especially in low-income countries [29]. This calls for further consideration of designing protocols for routine use of these modality of diagnosis in COVID-19 screening, where testing is still limited and or challenging.

## Conclusion

COVID-19 infection occurring in individuals with underlying or pre-existing medical conditions may become difficult to diagnose if it presents unusually with similar and overlapping clinical features to those conditions. This diagnostic challenge can be further aggravated in the presence of a problematic COVID-19 viral laboratory testing system. Thoracic imaging using chest CT scan is highly sensitive for screening patients with unclear clinical diagnosis either in the early or late stage of COVID-19 infection while chest X-ray is particularly useful during the late phase. The crucial role of this imaging modalities in COVID-19 screening becomes more apparent when real-time RT-PCR testing for COVID-19 is a challenge as can be found in many hospital settings of most low- and middle-income countries (LMICs).

### What is known about this topic

- COVID-19 remains a disease in evolution with more information coming through on daily basis to explain the disease epidemiology, pattern of presentation, case management and to guide public health control;
- Atypical presentations of COVID-19 are not uncommon;
- These atypical COVID-19 clinical presentations appear to be more common and follows a more severe course among patients with certain pre-existing medical conditions.

### What this study adds

- Atypical presentations of COVID-19 are not uncommon but may present a huge diagnostic challenge or difficulty with containment of intra-hospital disease transmission particularly where viral real-time PCR (RT-PCR) testing capabilities are difficult or limited. This is particularly important in resource-challenged hospital settings in low- and middle-income countries;
- The use of thoracic imaging especially chest computed tomographic scan in early evaluation of suspected COVID-19 cases, potentially has far-reaching clinical and public health benefits within a hospital setting, where and when possible and should be given strong considerations in situations where viral testing for COVID-19 is problematic and clinical presentation is atypical; regardless of the severity of the disease;
- Chest X-ray may not show classic radiological features of COVID-19 at the early stage of the disease. Therefore, the use of serial chest radiograph is advised in low-and middle-income countries where RT-PCR test kits or computed tomography (CT) are not readily available, bearing in mind the increasing diagnostic yield of chest X-ray as the disease progresses towards the late stage (averagely about day 8 - 12 from onset of symptoms).
